# Developing integrated care. Towards a development model for integrated care

**Published:** 2012-10-12

**Authors:** Mirella M.N Minkman

**Affiliations:** Program Leader Quality & Innovation in Elderly Care, Vilans, The National Center of Expertise in Long-Term Care, Catharijnesingel 47, 3503 RE Utrecht, The Netherlands E-mail: m.minkman@vilans.nl

## Introduction

The thesis explores the essential elements, implementation and developmental process of integrated care with a view to providing a quality management model for integrated care. Integrated care is required when a coordinated set of services is needed to cover the full range of client demands. The outcomes of this study add relevant information to our knowledge about integrated care and come together in the Development Model for Integrated Care (DMIC; in Dutch OMK: Ontwikkelingsmodel voor Ketenzorg). In addition the DMIC was empirically validated in practice.

## Integrated stroke and dementia care

The thesis starts with a study about the implementation of integrated stroke care and a multiple-case study in eight regional dementia care-provider networks. The study describes an improvement programme in 23 stroke services that was started because stroke services had not substantially improved despite the availability of best practices, evidence-based guidelines and quality criteria for stroke services. They formed multidisciplinary teams, analysed bottlenecks, set improvement aims, used rapid-cycle improvement and reviewed self-reported performance data. The topics worked on were length of stay and patient logistics, transfer of information between health care professionals, the improvement of after-care facilities and the implementation of thrombolysis treatment. Eighty-seven per cent of the teams improved their care significantly on at least one topic. About 34% of the teams have achieved significant improvements on all their aims. The study showed that a structured improvement programme can catalyse improvements in integrated care services. The dementia study describes and analyses an extensive case management approach in integrated dementia care in the Netherlands. Based on a literature study, a questionnaire was developed as a basis for 16 semi-structured interviews with the responsible (case) managers of the case management programmes. Although the eight programmes were developed independently there were similarities in approach. Factors for successful implementation are the expert knowledge of case managers, the investment in a strong provider network and coherent conditions for effective inter-organisational cooperation to deliver integrated care. Case managers favour a broad multi-task model during the whole care continuum and linkages with a multidisciplinary team and physicians. The programmes did not (as yet) assess the effects on client outcomes, service use and costs. When explored, caregiver and patient satisfaction were high. The (case) managers are convinced about the merits of case management intervention. Implementation of sustainable case management is considered as complex and time-consuming because of the many health care professionals and organisations involved with different interests and ways of financing. To facilitate implementation, a focus on joint responsibilities of the care providers involved is needed, together with incentives for collaborative contracts among financers like insurers and providers.

## Quality management model for integrated care

The next step was made to review the literature on the evidence for improving performance through the use of quality management models in health care and interventions based on those models. The European Foundation Quality Management Excellence model (EFQM), the Malcolm Baldrige Quality Award criteria (MBQA) and the Chronic Care Model (CCM) appear to be internationally and commonly used models with healthcare-specific versions and with assumed or proven relationships between the model components and improved results in health care. A systematic literature review in the Pubmed, Cochrane, and ABI databases was conducted. After selection 37 studies were included, 16 in the Excellence award model search and 21 in the CCM search. No Excellence Award model studies with controlled designs were found. For the CCM, one systematic review, one meta-analysis and six controlled studies were included. Seventeen studies reported one or more significant results. There is growing evidence that implementing interventions based on the CCM may improve process or outcome performances. The evidence for performance improvement by interventions based on the MBQA criteria and the EFQM Excellence model is more limited. Only a few studies include balanced measures on multiple performance dimensions. In only a few studies integrated care services are the domain subject of study. Considering the need for integrated (chronic) care, the further development of these or new models for guiding improvements in integrated care with their specific characteristics and context factors is crucial.

## A development model for integrated care

The next study in the thesis aimed to identify the elements and clusters of a quality management model for integrated care. First, a literature study was conducted which identified 101 elements of integrated care. Next an expert panel of 31 experts with experience working in research or integrated care programmes participated in a Delphi study. The experts commented and prioritised 175 elements in three rounds. In a session with the expert panel, Concept Mapping was used to cluster the elements. Multidimensional statistical analyses were applied to design the model. Based on criteria for inclusion and exclusion, 89 unique elements were determined after the three Delphi rounds, grouped into nine clusters. The clusters were labelled: ‘Quality care’, ‘Performance management’, ‘Inter-professional teamwork’, ‘Delivery system’, ‘Roles and tasks’, ‘Patient-centeredness’, ‘Commitment’, ‘Transparent entrepreneurship’ and ‘Result-focused learning’. The elements and clusters identified provide a basis for a comprehensive quality management model for integrated care. This model differs from other quality management models with respect to its general approach towards multiple patient categories and its broad definition of integrated care, ranging from acute to palliative care. The model furthermore highlights conditions for effective collaboration, such as commitment, clear roles and tasks and entrepreneurship. For integrated care practices, the model could serve as a basis for self or external evaluation of the integrated care service and provide inspiration for further improvement. The model also addresses nine interesting themes (the clusters) for further research on integrated care. For policy goals, the model could be used as a set of ‘organisational’ performance measures that can help in monitoring and stimulating balanced integrated care improvement.

## The development process of integrated care

Further on in the thesis a survey study on the developmental processes over time in integrated care is described to explore how local integrated care services are developed in the Netherlands. The process of taking integrated care to higher levels is described to only a limited extent in the literature and largely remains a black box. The research is based on an expert panel study followed by a two-part questionnaire. The 89 essential elements of integrated care were analysed in relation to the development process of integrated care. The study showed that integrated care development can be characterised by four developmental phases with different emphases that change over time. These phases were the initiative and design phase; the experimental and execution phase; the expansion and monitoring phase; and the consolidation and transformation phase. The results showed that in each of the phases different elements of integrated care could be identified as the most important ones. Overall the findings provided a descriptive model of the development process that integrated care services can undergo in the Netherlands. The study has important implications for integrated care services, which can use the model as an instrument to reflect on their current practices and identify improvement areas fitting their phase of development. To further assess the model’s value, empirical validation of our findings in practice is an important next step.

## Empirical validation of the Development Model for Integrated Care

One of the last important steps was the empirical validation of the 89 elements, nine clusters and four development phases of the Development Model for Integrated Care (DMIC) in integrated care practice. Based on the DMIC, a survey was developed for integrated care coordinators of three integrated care service settings in the Netherlands: stroke, acute myocardial infarct (AMI), and dementia. The survey focused on the relevance, implementation and plans of the elements, self-assessed development phases and factors that influence the development of the integrated care services. Eighty-four integrated care services participated in the study. The results indicate that the elements of the DMIC were rated as highly relevant in all three care settings. All participating services positioned themselves in one of the four phases, confirmed the phase descriptions and 93% confirmed that they recognised earlier phases. For the total group, the mean percentages of implemented elements were the highest in the ‘inter-professional teamwork’ and in the ‘roles and tasks’ clusters, while the lowest percentages were found in the ‘quality care’ and ‘performance management’ clusters. Timeline analyses showed that the older integrated care services had fewer plans for further implementation than the younger ones, as was presumed by the model. The self-assessment of development phases appeared to be complex, while the DMIC can be supportive in calculating the phase of development. The study also showed that elements corresponding to the earlier phases of the model were on average older, which indicates a certain pattern in development over time. Integrated care coordinators found that the DMIC helped them assess their integrated care and supported them in obtaining ideas for expanding their integrated care activities. Although the characteristics of the 84 participating integrated care services differed considerably, the results confirm that the clusters and phases and the vast majority of DMIC elements are relevant to all three groups. Support was therefore found for the conclusion that the DMIC can serve as a basis for a generic quality management tool for integrated care.

## Discussion and conclusions

**Figure 1.**
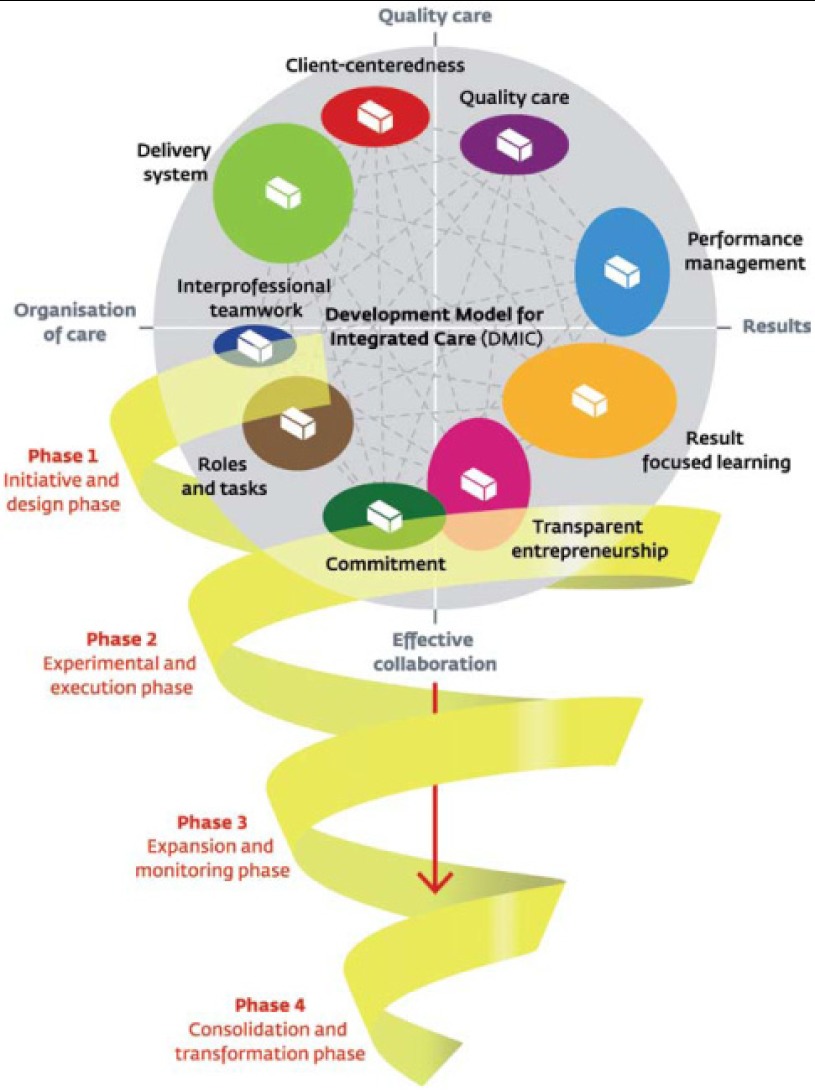
A development model for integrated care.

The thesis shows that the improvement and development process of integrated care is a long-term, multi-component process in which integrated care services cover a large range of activities. Quality management models are not frequently used in integrated care improvement and although there are many integrated care programmes, an evidence-based generic quality management model or a generic set of elements for integrated care were lacking. The 89 elements of integrated care as identified in this study, grouped into nine clusters, show that integrated care services do have generic components. Multiple aspects influence the dynamics and developmental process of integrated care services over time, but overall these processes can be conceptualised as phase-wise growth. The elements, clusters and four phases together formed the Development Model for Integrated Care (DMIC) (Figure 1). The model bears a resemblance to components of existing quality management models like the EFQM/MBQA models and the CCM, but has a wider focus on effective collaboration, commitment, learning, roles and tasks and entrepreneurship. Also it has a generic scope ranging from acute to chronic care. Another difference is the four development phases, which reflect the dynamics of integrated care. The DMIC was successfully validated in integrated stroke, AMI and dementia practices despite differences in client groups, size, focus, and the care providers involved. Phase-wise thinking is relatively new, but there is a certain order in practice which corresponds to our phases when elements are taken up. The variation found in practice regarding development and implementation is also the case when services are of the same ‘age’ and have the same contextual factors like legislation or financing. This shows that integrated care services have the opportunity to take up the challenge themselves.

## Implications for practice, policy and further research

For integrated care practices (coordinators, professionals and managers) the DMIC can be useful in assessing the current situation and guiding further improvement. The DMIC now forms the basis for a recently developed web-based self-assessment tool. When multiple participants use the tool in their integrated care service, consensus scores and improvement areas can be revealed, resulting in clarity about possible interventions appropriate to the particular phase of development. New studies show that the DMIC is also relevant for diabetes care, palliative networks, youth care, vulnerable elderly and autism care but more research on applying the DMIC within other client groups and for patients with multi-morbidities is recommended. Another suggestion is the application of the DMIC in other countries to assess the international relevance. A first study in Canada is initiated now, but more studies are needed. Lastly, we did reveal some of the dynamics of integrated care development but research on contextual and human factors (e.g. social relations, cultures, interests and power) would add value.

For policy-makers and financers this thesis provides information on stimulating the further development of integrated care. A recent pilot study with a health insurance company in the Netherlands made clear that the DMIC can also be supportive in purchasing integrated care. Another important issue is attention for the relationship between the organisation of integrated care, costs and its results. The aim of integrated care is after all to contribute to reducing fragmentation and to better outcomes, efficiency and costs. It seems plausible that further developed integrated care practices deliver better results, but evidence is needed. With the expansion of costs and the growing numbers of elderly and chronically (multi-morbid) patients, the question how to best and cost-effective organise our care is the main challenge in this decade.

## Articles published by the author

Minkman MNM, Schouten LMT, Huijsman R, van Splunteren PT. Integrated care for patients with a stroke in the Netherlands: results and experiences from a national Breakthrough Collaborative Improvement Project. International Journal for Integrated care [serial online] 2005 Mar 23;5. [cited August 30 2012]. Available from: http://www.ijic.org/. URN:NBN:NL:UI:10-1-100360.

Minkman MNM, Ligthart, Huijsman J. Integrated dementia care in the Netherlands: a multiple case study of case management programmes. Health and Social Care in the Community 2009 Sep;17(5):485–94.

Minkman MNM, Ahaus, Huijsman R. Performance improvement based on integrated quality management models: what evidence do we have? A systematic literature review. International Journal on Quality in Health Care 2007 Apr;19(2):90–104. Epub 2007 Feb 2.

Minkman MNM, Ahaus, Fabbricotti I, Nabitz Huijsman. A quality management model for integrated care: results of a Delphi and Concept Mapping study. International Journal on Quality in Health Care 2009 Feb;21(1):66–75. Epub 2008 Oct 22.

Minkman MNM, Ahaus, Huijsman J. A four-phase development model for integrated care services in the Netherlands. BMC Health Services Research 2009 Mar 4;9:42.

Minkman MNM, Vermeulen, Ahaus, Huijsman. The implementation of integrated care: the empirical validation of the development model for integrated care. BMC Health Services Research 2011;11(1):177.

Developing integrated care: a survey study to validate a four-phase development model for integrated care. (Submitted).

